# Role of interleukin-6 and interleukin-10 in morphological and functional changes of the blood–brain barrier in hypertriglyceridemia

**DOI:** 10.1186/s12987-023-00418-3

**Published:** 2023-03-07

**Authors:** Beáta Barabási, Lilla Barna, Ana Raquel Santa-Maria, András Harazin, Réka Molnár, András Kincses, Judit P. Vigh, Brigitta Dukay, Miklós Sántha, Melinda E. Tóth, Fruzsina R. Walter, Mária A. Deli, Zsófia Hoyk

**Affiliations:** 1 Institute of Biophysics, Biological Research Centre, Temesvári Krt. 62, Szeged, 6726 Hungary; 2 Doctoral School of Theoretical Medicine, University of Szeged, Tisza L. Krt. 109, Szeged, 6725 Hungary; 3 Present Address: Wyss Institute for Biologically Inspired Engineering at Harvard University, 3 Blackfan Circle, Boston, MA 02115 USA; 4 Institute of Biochemistry, Biological Research Centre, Temesvári Krt. 62, Szeged, 6726 Hungary

**Keywords:** Apolipoprotein B-100, Blood–brain barrier, Brain microvessel, Hypertriglyceridemia, Interleukin-6, Interleukin-10, Tight junction, P-glycoprotein, Claudin-5, Aquaporin-4, Glia

## Abstract

**Background:**

Hypertriglyceridemia is closely linked to atherosclerosis related inflammatory processes and blood–brain barrier (BBB) dysfunction. Using apolipoprotein B-100 (APOB-100) transgenic mice, an animal model of chronic hypertriglyceridemia, we analyzed BBB function and morphology in vitro and ex vivo. Our objective was to determine which BBB characteristics are produced mainly by interleukin (IL)-6, an atherosclerosis promoting cytokine, and whether these actions can be antagonized by IL-10, an anti-inflammatory cytokine.

**Methods:**

Brain endothelial and glial cell cultures and brain microvessels were isolated from wild type (WT) and APOB-100 transgenic mice and were treated with IL-6, IL-10 and their combination. First, IL-6 and IL-10 production was measured in WT and APOB-100 microvessels using qPCR. Then functional parameters of endothelial cell cultures were analyzed and immunocytochemistry for key BBB proteins was performed.

**Results:**

IL-6 mRNA levels were higher in brain microvessels than in brain parenchyma of APOB-100 transgenic mice. Transendothelial electric resistance and P-glycoprotein activity were lower, and paracellular permeability was higher in cultured APOB-100 brain endothelial cells. These features were sensitive to both IL-6 and IL-10 treatments. A decreased P-glycoprotein immunostaining was measured in transgenic endothelial cells under control conditions and in WT cells after treating them with IL-6. This effect was antagonized by IL-10. Changes in immunostaining for tight junction proteins were observed after IL-6 exposure, which were in part antagonized by IL-10. In glial cell cultures an increase in aquaporin-4 immunolabeling in the transgenic group and an increase in microglia cell density in WT glia cultures was detected after IL-6 treatment, which was antagonized by IL-10. In isolated brain microvessels a decrease in P-glycoprotein immunolabeled area fraction was measured in APOB-100 microvessels under control conditions and in WT microvessels after every cytokine treatment. ZO-1 immunolabeling showed characteristics similar to that of P-glycoprotein. No change was seen in claudin-5 and occludin immunoreactive area fractions in microvessels. A decrease in aquaporin-4 immunoreactivity was measured in WT microvessels treated by IL-6, which was antagonized by IL-10.

**Conclusion:**

IL-6 produced in microvessels contributes to BBB impairment observed in the APOB-100 mice. We showed that IL-10 partly antagonizes the effects of IL-6 at the BBB.

**Supplementary Information:**

The online version contains supplementary material available at 10.1186/s12987-023-00418-3.

## Background

Hyperlipidemia, including high levels of low-density lipoprotein (LDL) particles, is a common pathological condition. It is closely linked to the development of atherosclerosis [1], because LDL particles, which transport cholesterol esters in the blood, are susceptible to oxidative processes in the subendothelial space [2, 3]. Oxidized LDL induces expression of cell adhesion molecules on endothelial cell surfaces, leading to monocyte recruitment. Monocytes, in turn, enter the subendothelial space where they progressively differentiate into macrophages, which take up oxidized LDL and subsequently are converted into foam cells. Smooth muscle cells of the arterial wall may also accumulate modified lipids contributing to both conversion to foam cell and fibrous cap formation [4]. Adhesion molecules and chemokines are involved not only in monocyte recruitment but also participate in guiding T lymphocytes into atherosclerotic plaques. The immune cells present in atherosclerotic lesions interact with each other and secrete a broad range of cytokines with pro- and anti-inflammatory effects [4].

Macrophages and smooth muscle cells in atherosclerotic lesions produce interleukin (IL)-6, a pleiotropic cytokine, which is associated with endothelial dysfunction [5–7]. Vascular cells may promote inflammatory processes by synthesizing tumor necrosis factor (TNF)-α, IL-1β, IL-6, IL-8 and IL-15, and may exert anti-inflammatory action mainly by transforming growth factor-β production. Another cytokine playing an anti-inflammatory role in atherosclerosis is IL-10, which is primarily produced by macrophages and T and B lymphocytes [8] and in very small quantities by brain capillary endothelial cells [9]. Due to the prolonged expression of a wide repertoire of pro-inflammatory cytokines, atherosclerosis is considered a chronic inflammatory disease [8]. Hyperlipidemia is associated with systemic inflammation even without cardiovascular pathologies. Patients with high triglyceride levels have an increased capacity to produce TNFα and IL-6 [10]. Systemic inflammation also affects the brain and its vasculature, and damages their functions [11].

Capillaries in the central nervous system represent a dynamic interface, known as the blood–brain barrier (BBB), between the brain parenchyma and the circulating blood. The cellular components of the mature BBB are non-fenestrated endothelial cells, pericytes and astroglial endfeet [12]. Endothelial cell–cell interactions and, consequently, paracellular permeability is controlled by endothelial tight junctions made of integral transmembrane proteins (occludin and predominantly claudin-5), adaptor cytoplasmic proteins (ZO-1, -2), and by adherens junction proteins [12]. The exchange of solutes across the BBB occurs in a controlled manner via transcellular transport systems, including carrier-mediated and receptor-mediated transport, adsorptive transcytosis and active efflux transport [13, 14]. The chemoprotection of the brain by the efflux of potentially toxic lipophilic/amphiphilic molecules is mediated by ATP-binding cassette transporters, like the multidrug resistance transporter P-glycoprotein (P-gp/ABCB1), which shows reduced expression in systemic inflammation [15] and impaired function in neurodegenerative diseases [16].

BBB properties highly depend on the cross-talk between brain capillary endothelial cells, astroglial processes and pericytes. In the adult, the BBB is stabilized by perivascular cells [17]. Astroglial processes develop microvascular endfeet that cover the vasculature forming part of the BBB [18]. These astrocytic perivascular endfeet contain the water channel aquaporin 4 (AQP4) in tenfold higher densities than non-endfoot astrocyte membranes [19]. It permits bidirectional water flow between brain and blood driven by osmotic gradients contributing to maintain ion and volume homeostasis under normal conditions. During pathologic events, like neuroinflammation, however, AQP4 protein expression is decreased in the perivascular endfeet indicating impairment of BBB function [19].

Among the brain perivascular cells astrocytes and pericytes are able to produce cytokines, including TNFα and IL-6 under control conditions in culture [20]. Brain endothelial cells, in contrast, do not produce TNFα nor IL-6 in a healthy microenvironment, but they react to challenges, such as LPS stimulation, which induces endothelial TNFα and IL-6 production [20]. Astroglia and endothelial cells may participate not only in IL-6 production, but their function can also be modulated by IL-6, since these cells express IL-6 receptors [21–24].

Moreover, astroglia and endothelial cells can respond to the anti-inflammatory cytokine IL-10, too [25]. Microglia are located also in the perivascular niche, between the vascular wall and astrocytic endfeet. Microglia can also make direct contacts with the cells of the BBB including endothelial cells and regulate cerebral blood flow [26]. Microglia cells express receptors for various cytokines produced by cellular components of the BBB during atherosclerosis, including IL-6 [21], which may contribute to microglia activation. Microglia reactions, in turn, may involve release of pro-inflammatory cytokines TNFα and IL-1β leading to BBB impairment [27]. Pro-inflammatory cytokines including TNFα and IL-6 can be produced not only within the neurovascular unit, but they can reach the brain vasculature originating from peripheral inflammatory conditions, resulting in increased BBB permeability [11]. Microglial production of pro-inflammatory cytokines may be inhibited by IL-10 [28], an anti-inflammatory cytokine synthesized by immune cells infiltrating atherosclerotic plaques [8].

The transgenic mouse line overexpressing the human apolipoprotein (APO) B-100 protein is one of the well characterized animal models of human atherosclerosis [29]. APOB-100 is an essential component of lipoprotein particles transporting triglycerides. Therefore, these transgenic mice are characterized by chronic hypertriglyceridemia. In our previous work we demonstrated that these animals also show cerebrovascular pathologies, including increased permeability of brain capillaries for a small molecular marker, decreased expression of genes coding tight junction (TJ) proteins claudin-5, occludin and zonula occludens-1 (ZO-1) protein and the efflux transporter P-gp, and an increased expression of the glial endfeet marker AQP4 gene [30]. We could also detect alterations related to neuroinflammation in the APOB-100 transgenic mice such as a significant increase in the expression of TNFα and nuclear factor (NF)-κB genes in the cerebral cortex. At protein level, we reported a decrease in immunofluorescence intensity of P-gp and the astroglia marker GFAP [29, 30]. These findings suggest that the inflammatory cytokine TNFα may have a role in BBB dysfunction in this mouse model. However, little is known on the effects of other cytokines related to atherosclerosis on the expressional changes of BBB molecular markers and BBB functions.

We hypothesize that IL-6, a pleiotropic pro-inflammatory cytokine playing key roles in atherosclerosis, may significantly contribute to BBB dysfunction, too. In addition, the anti-inflammatory cytokine IL-10, acting as an IL-6 antagonist, may prevent at least some of the alterations related to IL-6 treatment and to BBB damage. Therefore, in the current study our aim was to investigate the effects of IL-6 either alone or in combination with IL-10 on BBB characteristics in wild-type (WT) and APOB-100 transgenic mice. We analyzed BBB function and morphology following IL-6 and IL-10 treatments in primary brain endothelial cell cultures and cerebral microvessels isolated from WT and APOB-100 transgenic mice. BBB functional characterization was carried out by measuring transendothelial electrical resistance (TEER), paracellular permeability and P-gp activity. The functional assays were completed with analysis of the immunostaining patterns of endothelial TJ proteins, the efflux pump P-gp, and the glial endfeet marker AQP4 in isolated cerebral microvessels and in primary cell cultures, respectively. The astroglial cell marker GFAP and the microglial marker Iba-1 immunolabelings were visualized in glial cell cultures derived from both genotype to examine glial reactions to IL-6 and IL-10 treatments.

## Methods

### Materials

All reagents were purchased from Merck (Budapest, Hungary) except for those specifically mentioned.

### Animals

Mice were housed in groups of two to three under standard conditions (24 °C, 12 h light–dark cycle) with food and water available ad libitum. Animals were maintained on a regular rodent chow diet. The APOB-100 mouse strain overexpressing the human APOB-100 protein was previously established by the group of Miklós Sántha [31]. This APOB-100 mouse strain was bred and maintained on a C57BL/6 genetic background in a hemizygous form. Breeding of the transgenic mouse strain was approved by the regional Animal Research Ethics Committee (Csongrád county, Hungary; project license: XVI./2724/2017). All animals were handled in accordance with approved procedures as defined by the EU Directive 2010/63/EU. In order to determine the genotype of hemizygous APOB-100 animals and wild-type littermates, DNA from tail biopsies of 2-, or 10-day-old pups was purified, and the presence of the transgene was detected by PCR, using primers for the 5’ promoter region of the human APOB-100 gene.

### Cell cultures

#### Brain endothelial cell cultures

The primary cultures of brain endothelial cells (BECs) were prepared from 6–7-month-old WT and APOB-100 transgenic mice as described in detail by Lénárt et al. [32]. 3 male and 3 female mice were used in both the WT (n = 6) and the APOB-100 group (n = 6) for each isolation. Forebrains were collected in ice-cold sterile PBS; meninges were removed, grey matter was minced by scalpel into 1 mm^3^ pieces and digested with 10 mg/ml collagenase II and 1 mg/ml DNase I in Dulbecco’s modified Eagle’s medium (DMEM)/F12 for 50 min at 37 °C. Microvessels were separated from myelin containing elements by centrifugation (1000×*g*, 20 min) in 20% bovine serum albumin (BSA)-DMEM and further digested with 10 mg/ml collagenase-dispase (Roche, Basel, Switzerland) and 1 mg/ml DNase I in DMEM/F12 for 35 min at 37 °C. Then they were washed twice in DMEM/F12 before plating on collagen type IV and fibronectin-coated (100 µg/ml each) dishes, 6 well plates (Corning Costar Co., Lowell, MA, USA) or cell culture inserts (Transwell clear, 1 cm^2^; pore size of 0.4 μm; Corning Costar Co.). Cultures were maintained in DMEM/F12 supplemented with 15% plasma-derived bovine serum (PDS; First Link, Wolverhampton, UK), 1 ng/ml basic fibroblast growth factor (Roche) and 100 μg/ml heparin. During the first 2 days, the culture medium contained puromycin (4 μg/ml) in order to selectively remove P-glycoprotein-negative contaminating cells [33]. Cultures reached confluency within a week and were used for experiments. To induce BBB characteristics, BECs were co-cultured with mouse astroglial cells. The resulting double co-culture model was used for permeability studies and transendothelial electrical resistance measurements [34, 35].

#### Mixed glial cell cultures

Primary mouse glial cells were isolated and cultured as described in our earlier publication [32]. Briefly, 3 male and 3 female mice were used in both the WT (n = 6) and the APOB-100 group (n = 6) for each isolation. Forebrains were obtained from 3 or 4-day-old WT and APOB-100 transgenic mice and placed into ice-cold PBS. Meninges were removed and little pieces of cortices were pipetted into 50-ml tubes and then the tissue was mechanically dissociated by using a long and thin needle (21G 4 ¾, Braun, Germany). Isolated cells were plated onto uncoated T25 flasks (Corning Costar Co.) and cultured in low-glucose DMEM (Thermo Fisher Scientific, Waltham, Massachusetts, USA), which contained 10% fetal bovine serum (Sera Plus, Pan Biotech, Aidenbach, Germany) and gentamycin (50 μg/ml). Glial cells were cultured until confluency with medium change every 2 days. Then glial cells were seeded onto poly-l-lysine-coated coverslips placed into 24-well plates and cultured with medium change every 3 days. Cultures reached confluency in 5–7 days. Then they were treated with cytokines, fixed and immunostained. Confluent cell layers consisted of 57% astrocyte and 43% microglia in WT, and 49% astrocyte and 51% microglia in APOB-100 glia cultures.

### Brain microvessel isolation

Cortical microvessels were isolated from the brain of 6–7-month-old animals, as described earlier [36]. 3 male and 3 female mice were used in both the WT (n = 6) and the APOB-100 group (n = 6) for each isolation. The forebrains of APOB-100 or WT mice were collected in ice-cold sterile phosphate buffered saline (PBS). Meninges were taken off by rolling brains on a sterile wet filter paper. White matter and the choroid plexus were removed and the tissue was cut into 1 mm^3^ pieces by scalpels. Samples then were homogenized in ice-cold Ringer-HEPES buffer (4 ml/g of tissue), and the resulting homogenates were centrifuged at 2000 g for 10 min. After centrifugation the microvessel enriched pellets were resuspended in 17.5% dextran (64–76 kDa) in Ringer-HEPES (118 mM NaCl, 4.8 mM KCl, 2.5 mM CaCl_2_, 1.2 mM MgSO_4_, 5.5 mM D-glucose, 10 mM HEPES, pH 7.4) and centrifuged at 4 °C, 4400*g* for 15 min. The resulting pellets were suspended in 2 ml Ringer-HEPES buffer containing 1% BSA, while the supernatants were collected and centrifuged once more. The resulting pellets were pooled and passed through nylon meshes with 100 µm and 20 µm pore size. The microvessels retained by the mesh with 20 µm pore size were washed off with 15 ml buffer and centrifuged at 4 °C, 1000*g* for 10 min. After a second wash in 10 ml buffer (4 °C, 700*g*, 5 min), microvessels were immediately treated with cytokines.

### RNA isolation and quantitative real-time PCR

For RNA isolation, forebrains of 8 WT and 8 APOB-100 transgenic mice were used (7 months old; 4 male and 4 female animals/group). Hemispheres of the brains were separated and the left hemispheres were further divided into hippocampal and cortical regions. Right hemispheres were pooled and were used for microvessel isolation as described above. Samples were stored in RNA-later solution (Invitrogen, Life Technologies, USA) at − 80 °C until use. Total RNA was isolated from the hippocampal and cortical brain regions and from microvessel samples using an RNA and protein purification kit (Macherey–Nagel, Düren, Germany) according to the manufacturer’s instructions. High Capacity cDNA Reverse Transcription Kit (Thermo Fisher Scientific, Waltham, Massachusetts, USA) was used to convert mRNA samples to cDNA. Each reaction mixture contained 15 µL RNA sample (1000 ng in the case of hippocampal and cortical samples, 350 ng for microvessels), 1.5 µl reverse transcriptase, 3 µl primer, 1.2 µl dNTP, 3 µl buffer, 6.3 µl RNase-free water. Parameters for the reverse transcription program were the following: incubation at 25 °C for 10 min, reverse transcription at 37 °C for 2 h, and inactivation at 85 °C for 5 min (using MJ Mini—Personal Thermal Cycler, BioRad). The cDNA product was finally diluted 1:20, and was used as a template in the qPCR reaction. For the qPCR reaction, 10 µl cDNA, 1 µl (250 nM final) primer mix (forward + reverse), and 10 µl Power SYBR Green PCR Master Mix 2x (Thermo Fisher Scientific, Waltham, Massachusetts, USA) were mixed. Each reaction was performed in a total volume of 20 µl, and was run on a RotorGene 3000 instrument (Qiagen, Hilden, Germany) with the following settings: heat activation at 95 °C for 10 min; followed by 40 cycles of denaturation at 95 °C for 15 s, annealing at 60 °C for 60 s*.* Melting curve analysis was performed between 50 and 95 °C to verify the specificity of the amplification. Primer sequences used in qPCR reactions are listed in Additional file 1: Table S1, of which the mouse *Gapdh* (for hippocampal and cortical samples) and *Actb* (for microvessel samples) genes served as an internal control for normalization. Relative gene expression levels were calculated using the ΔΔCt method.

### Cell viability assay

Kinetics of the viability of BECs was observed by a real-time impedance measurement (RTCA-SP, Agilent, Santa Clara, CA, USA) as described previously [37]. Impedance correlates linearly with cell number, adherence, growth and viability. BECs were grown on golden electrodes of 96-well E-plates (Agilent) in a CO_2_ incubator at 37 °C for 5 days. Then the cells were treated with cytokines at 10 or 50 ng/ml concentration in 3 combinations: IL-6, IL-10 and IL-6 + IL-10. Effects of treatments were monitored for 24 h, as shown for IL-6 in Additional file 1: Fig. S1.

### Cytokine treatments

Treatment of primary BECs and glial cells was carried out in a suitable culture medium for each cell type for a maximum treatment period of 24 h. Cortical microvessels were treated immediately after isolation for a period of 1 h. For these treatments, both IL-6 and IL-10 were used at 50 ng/ml concentration.

### Evaluation of barrier integrity

#### Transendothelial electrical resistance measurement

Transendothelial electrical resistance (TEER) representing the permeability of tight junctions for sodium ions in culture conditions was measured by an EVOM resistance meter (World Precision Instruments Inc., Sarasota, FL, USA) using STX-2 electrodes, and expressed relative to the surface area of the endothelial monolayer (Ω × cm^2^) as described in our earlier publication [32]**.** The background TEER value of inserts without cells (21 Ω × cm^2^) was subtracted from the measured values.

#### Permeability measurement

Permeability tests using the small molecular marker sodium fluorescein (SF, MW = 376 Da) were carried out on an in-contact type double co-culture BBB model with primary astroglia when high TEER values were recorded. After applying IL-6, IL-10 and IL-6 + IL-10 in combination (both cytokines at 50 ng/ml concentration) for 24 h in the luminal compartment, the test was conducted as described previously [37]. The concentration of the SF marker molecule in samples from the upper and lower compartments was determined with a microplate reader (excitation at 440 nm, emission at 525 nm; BMG Fluostar Optima; BMG Labtech, Ortenberg, Germany). Flux across coated, cell-free inserts was also measured. Endothelial permeability coefficients (P_e_) were calculated from clearance values of tracers as described in detail in our previous publication [32].

#### P-glycoprotein activity measurement

P-glycoprotein (P-gp/ABCB1) activity was determined by measuring the cellular accumulation of the efflux pump ligand rhodamine 123 (R123). BECs were seeded in 24-well plates and incubated in 10 µM R123 in Ringer-HEPES for 1 h at 37 °C after cytokine treatments. Cyclosporine A (1.6 µM), which blocks P-gp and breast cancer-resistant protein/ABCG2 was used as a reference inhibitor molecule. Following cytokine treatments and incubation with R123, BEC monolayers were washed 3 times with ice-cold PBS, then solubilized in 0.1 M NaOH. Fluorescence intensity indicating intracellular R123 concentration was measured in a 96-well plate (Fluostar Optima; excitation: 485 nm, emission: 520 nm).

### Immunocytochemistry

The immunostaining pattern of claudin-5, occludin, ZO-1 and P-gp was studied in primary BECs isolated from WT and APOB-100 transgenic mice. Cells were fixed with ice-cold acetone-methanol for 2 min, then non-specific binding was blocked with 1% BSA in PBS at room temperature during 1 h. Cells were incubated with primary antibodies shown in Additional file 1: Table S2 overnight at 4 °C, which was followed by a 1 h incubation with the corresponding secondary antibodies (A594-conjugated donkey anti-rabbit, A488-conjugated goat anti-mouse (Thermo Fisher Scientific, MA, USA) and Cy3-labeled sheep anti-rabbit, as shown in Additional file 1: Table S2). Cellular nuclei were stained with Hoechst 33,342 (Thermo Fisher Scientific) at a concentration of 1 µg/ml. The samples were mounted (Fluoromount-G; Southern Biotech, AL, USA), then examined using a Spinning Disk Confocal Microscope (Zeiss, Germany).

Astroglia and microglia were immunolabeled to visualize GFAP, S100B, AQP4 and Iba-1 expression, respectively. Glial cells were fixed with 3% paraformaldehyde and permeabilized with 0.2% Triton X-100 in PBS for 10 min. Non-specific binding of antibodies was blocked with 3% BSA in the case of S100B + AQP4 co-staining, and with 2% normal horse serum and 5% normal goat serum for Iba-1 + GFAP co-labeling. Glial cells were incubated with primary antibodies (Additional file 1: Table S2) overnight at 4 °C, followed by incubation with the corresponding secondary antibodies for 1 h (A488-labeled donkey anti-goat (Thermo Fisher Scientific), DyLight 549-conjugated goat anti-mouse (Jackson ImmunoResearch Europe Ltd., Cambridgeshire, UK), A594-conjugated donkey anti-rabbit (Thermo Fisher Scientific), as shown in Additional file 1: Table S2. Hoechst dye 33,342 was used for nuclear staining at a concentration of 1 µg/ml. After mounting the samples (Fluoromount-G; Southern Biotech), the immunoreactivity was examined using a Leica TCS SP5 confocal laser scanning microscope (Leica Microsystems, Germany).

Isolated brain microvessels were fixed with 3% paraformaldehyde immediately after cytokine treatment, and the expression pattern of key BBB proteins claudin-5, occludin, ZO-1, P-gp and AQP4 was analyzed using immunocytochemistry. Microvessels were permeabilized and non-specific binding was blocked with 0.2% Triton X-100 and 2% normal serum in PBS for 10 min. Then microvessels were incubated overnight at 4 °C with primary antibodies at dilutions shown in Additional file 1: Table S2. The next day, microvessels were incubated for 50 min (Additional file 1: Table S2) with the corresponding secondary antibodies, i.e. A594-conjugated donkey anti-rabbit and A488-conjugated goat anti-mouse (Thermo Fisher Scientific), as shown in Additional file 1: Table S2. Cellular nuclei were stained with Hoechst 33342 (Thermo Fisher Scientific) at a concentration of 1 µg/ml. Between incubations microvessels were washed three times with PBS. The staining patterns were examined with a Spinning Disk Confocal Microscope (Zeiss, Germany).

### Image analysis

Fluorescence intensity of immunolabelings in BEC cultures was quantified using the ImageJ program. The background intensity was subtracted from integral intensity values of immunofluorescent signals. The resulting immunofluorescence values were normalized to the number of pixels showing immunolabeling. The number of images analyzed was n = 5–15/treatment group for each staining.

Cell density was determined by nucleus number/10000 µm^2^. In the case of mixed glial culture, astroglia and microglia nuclei were counted separately. The WT control was considered 100%, all the other treatment groups and transgenic groups were normalized to it. In order to assess the ratio of microglia and astroglia in the mixed culture, their cell numbers in each picture were compared and their ratio was calculated (n = 8–10).

The immunolabeling in isolated microvessels was quantified by calculating the area fraction of immunostained structures in microvessels using Matlab software. Images were divided into smaller fractions by an expert in immunohistochemistry using freehand drawing in order to get images containing one immunolabeled microvessel only and discard signals coming from tissue debris. Then, low threshold was set for the binarization of the immunolabeling to highlight the whole surface of the microvessel. The pixel numbers of these segments gave the area of the microvessels on each image. Next, the global threshold of the specific immunostaining was determined using Otsu’s method, resulting in binary images of the immunolabeled structures [38]. The pixel numbers of these binary images represented the area of the labeled structures. Each area of immunostained structure was normalized to the corresponding total microvessel area. The number of images analyzed was n = 5–15/treatment group for each staining.

### Statistical analysis

GraphPad Prism 5.0 software (GraphPad Software Inc. LaJolla, CA, USA) was used for statistical analysis. Gaussian distribution of the data was tested with the Kolmogorov–Smirnov normality test. Data showing Gaussian distribution were analyzed with two-way analysis of variance followed by Bonferroni post hoc test. Data showing no Gaussian distribution were analyzed with Kruskal–Wallis and Dunn’s multiple comparison tests. The level of statistical significance was taken as p < 0.05. Results are presented as means ± SEM.

## Results

### Cell cultures

**Fig. 1:**
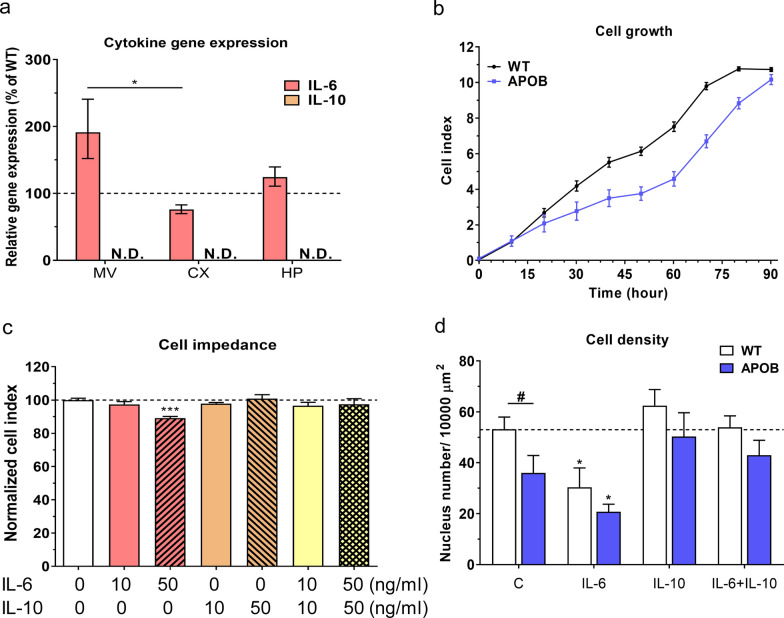
Analysis of cytokine gene expression, cell growth, effective cytokine concentration and cell density. **a** IL-6 and IL-10 gene expression levels measured with qPCR in isolated brain microvessels (MV), cortex (CX) and hippocampus (HP) of wild type (WT) and apolipoprotein B-100 (APOB-100) transgenic mice. IL-10 levels are under the detection limit. **b** Cell viability assay on primary brain microvascular endothelial cells isolated from WT and APOB-100 transgenic mice. **c** Concentration dependent effects of IL-6, IL-10 and IL-6 + IL-10 on WT endothelial cell viability. **d** Density of primary microvascular endothelial cells isolated from WT and APOB-100 transgenic mice following IL-6, IL-10 and IL-6 + IL-10 cytokine treatments at 50 ng/ml concentration. #: significant change between WT and APOB-100 cells (p < 0.05); *: significant change due to cytokine treatment compared to the control group with the same genotype (p < 0.05)

In our first series of experiments expression levels of genes coding IL-6 and IL-10 were measured in isolated brain microvessels and in brain tissue using qPCR. IL-6 showed a significantly higher expression in cerebral microvessels compared to brain cortex in APOB-100 transgenic mice. IL-10 expression was under the detection limit in each sample (Fig. 1a). The cell growth kinetics of brain endothelial cells from WT and APOB-100 animals was analyzed by impedance measurements on primary cultures of these cells. Based on the growth curves a slower proliferation rate in transgenic endothelial cell cultures was measured (Fig. 1b). Next, the effective concentration of cytokines on primary brain endothelial cell cultures was determined. IL-10 showed no harmful effects in either concentration, but IL-6 treatment resulted in a decrease in normalized cell index at a concentration of 50 ng/ml (Fig. 1c). At the higher concentration an early effect of IL-6 with steady decrease until 24 h was measured by impedance kinetics (Additional file 1: Fig. S1). This effect was antagonized by IL-10 when applied in combination with IL-6 (Fig. 1c). The density of primary brain endothelial cell cultures was significantly decreased in transgenic cultures compared to WT cells under control conditions. Following treatment of endothelial cell cultures with IL-6 (50 ng/ml) a significant decrease in cell density was seen in both genotypes. However, when IL-6 was applied in combination with IL-10 no change was observed in cell density (Fig. 1d).

**Fig. 2:**
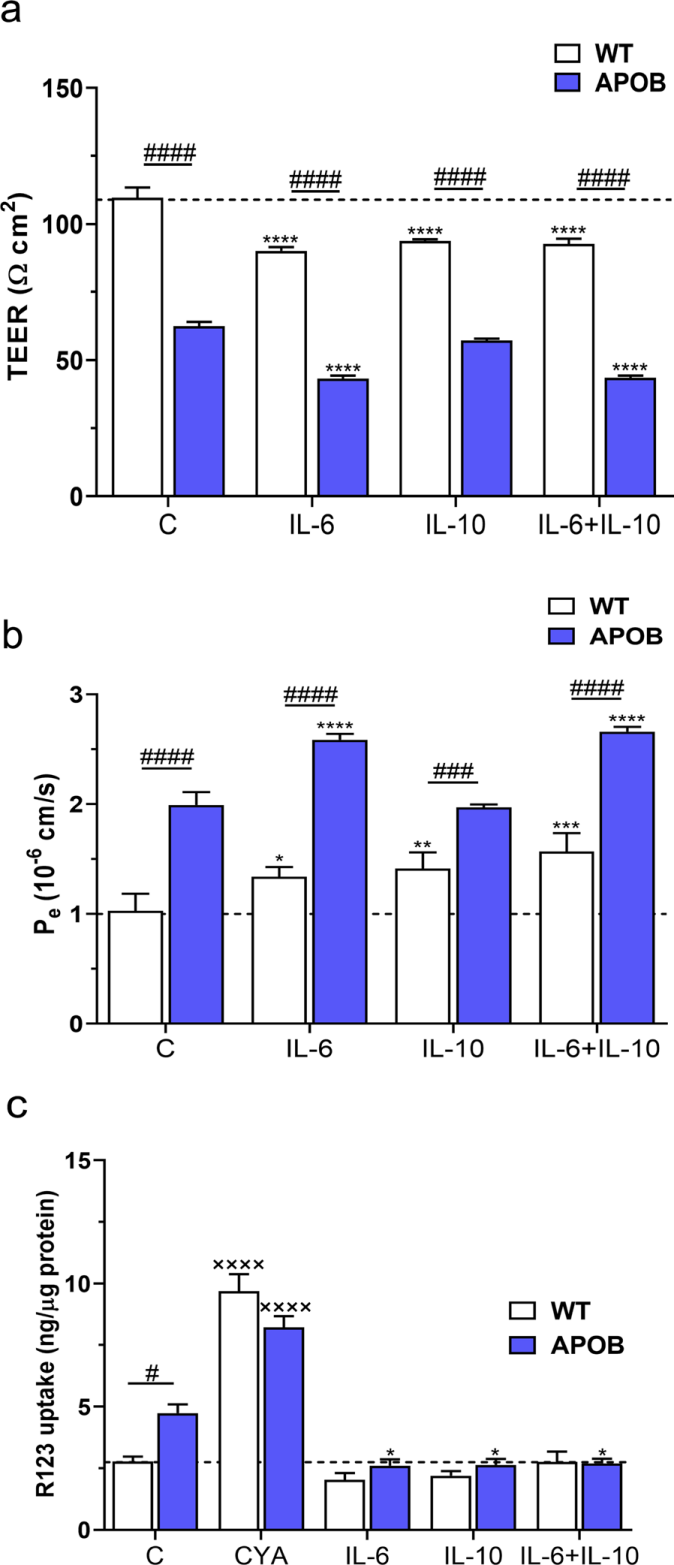
Effects of cytokine treatments on the function of brain microvascular endothelial cells. **a** TEER values in wild type (WT) and apolipoprotein B-100 (APOB-100) endothelial cell cultures treated with Il-6, IL-10, and their combination. **b** Permeability for sodium fluorescein (SF) in WT and APOB-100 endothelial cell cultures under control conditions and following cytokine treatments. **c** Activity of the efflux pump P-glycoprotein in WT and APOB-100 endothelial cell cultures. R123: rhodamine 123; CYA: cyclosporin A. #: significant change between WT and APOB-100 cells (p < 0.05); *: significant change due to cytokine treatment compared to the control group with the same genotype (p < 0.05); xxxx: significant change due to cyclosporin A (p < 0.0001)

Brain endothelial cell function was monitored by TEER and permeability measurements and P-gp activity assays using co-culture BBB models. APOB-100 transgenic endothelial cells co-cultured with APOB-100 transgenic glial cells showed a decrease in TEER compared to the WT BBB co-culture model both with and without cytokine treatments. IL-6 resulted in more decreased TEER values in transgenic cells, which was not inhibited by IL-10. However, IL-10 applied alone did not change TEER compared to that measured in transgenic cells under control conditions. In WT cells, in contrast, a significant decrease in TEER was detected following each cytokine treatment compared to control values. (Fig. 2a). The permeability for the small molecular marker SF was increased in APOB-100 endothelial cells compared to WT cells in each experimental group. Following IL-6 treatment an increase in paracellular permeability was detected in WT cells and a further increase was observed in transgenic cells. IL-10 applied alone did not change permeability values in transgenic cells and did not antagonize IL-6 effects. Interestingly, IL-10 resulted in an increase in paracellular permeability in WT cells, which was not antagonized by IL-6 (Fig. 2b). Another parameter which characterizes brain endothelial cell function is P-gp activity. A decreased P-gp activity, reflected in elevated cellular uptake of R123, was measured in brain endothelial cells isolated from APOB-100 transgenic mice compared to that detected in WT cells without cytokine treatment. This activity showed no change in WT cells following either cytokine application. Transgenic endothelial cells, in contrast, reacted to each cytokine applied with an increase in P-gp activity compared to activity levels seen in transgenic cells under basal conditions (Fig. 2c).

In our next series of experiments morphological changes in WT and APOB-100 primary brain endothelial cells cultures were studied under control conditions and following cytokine treatments. The morphological analysis focused on immunostaining patterns of the efflux pump P-gp and of TJ proteins claudin-5, occludin and ZO-1.

**Fig. 3:**
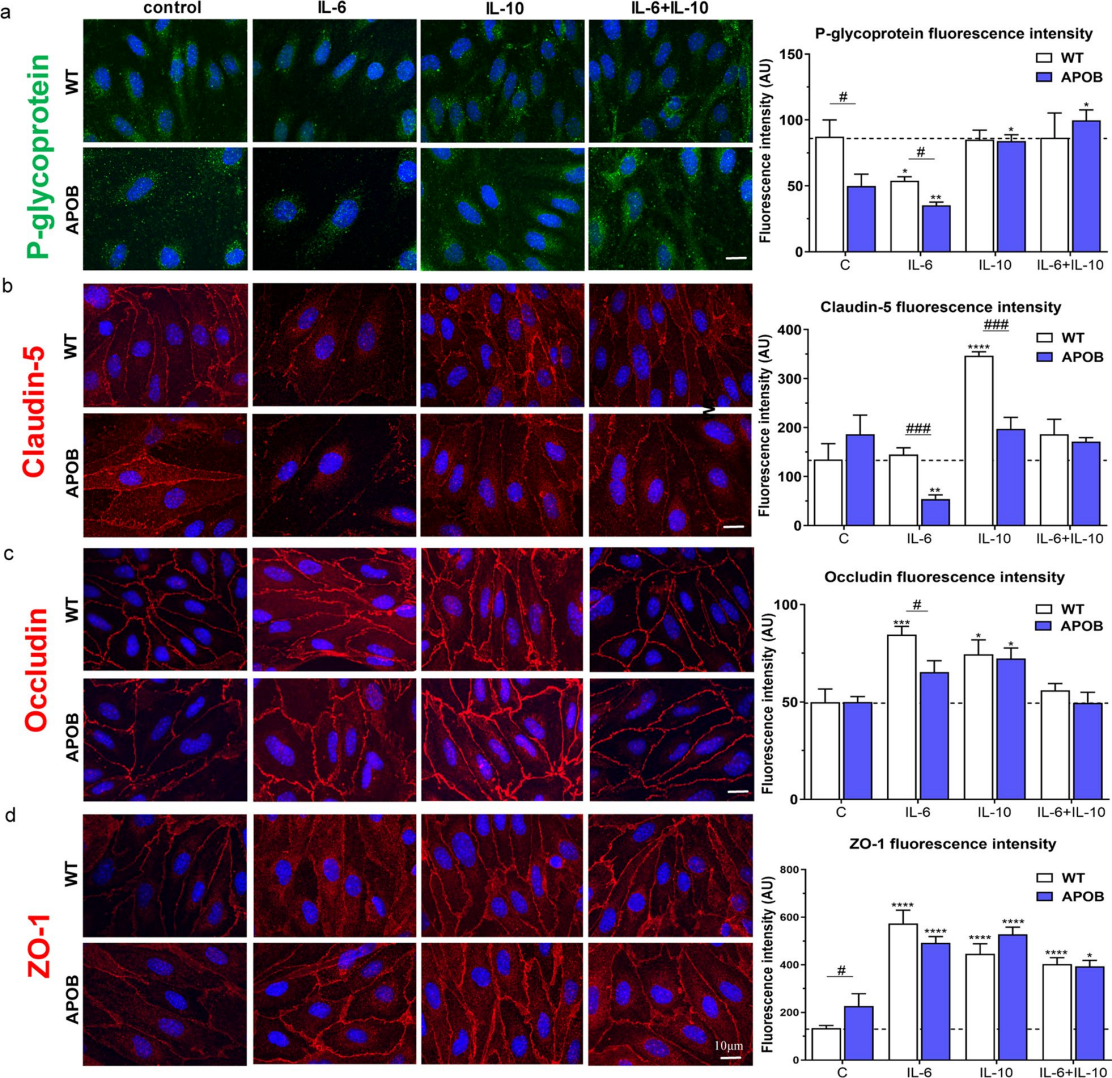
Immunostaining and fluorescence intensity of key BBB proteins in endothelial cell cultures following cytokine treatments. **a** P-glycoprotein, **b** claudin-5, **c** occludin, **d** zonula occludens protein-1 (ZO-1) immunolabeling of wild type (WT) and apolipoprotein B-100 (APOB-100) brain endothelial cell cultures. AU: arbitrary unit. #: significant change between WT and APOB-100 cells (p < 0.05); *: significant change due to cytokine treatment compared to the control group with the same genotype (p < 0.05)

P-gp immunolabeling was characterized by a patchy staining pattern in the cytoplasm of endothelial cells from both genotypes (Fig. 3a). The fluorescence intensity of P-gp immunolabeling showed a decrease in transgenic cells compared to WT cells under control conditions. The intensity of P-gp immunostaining was altered due to cytokine application. Brain endothelial cells isolated from WT mice showed a decrease in P-gp immunofluorescence after IL-6 treatment, resulting in intensity values similar to those measured in APOB cells under control conditions. This effect was antagonized by IL-10. No change in P-gp fluorescence intensity was observed in WT endothelial cells when IL-10 was applied alone. Transgenic cells exhibited a decrease in P-gp immunofluorescence intensity following treatment with the pro-inflammatory cytokine IL-6 and an increase in P-gp intensity after the anti-inflammatory cytokine IL-10 exposure, compared to control transgenic cells. IL-10 applied in combination with IL-6 antagonized the effects of IL-6 in both genotypes and resulted in immunofluorescence intensity values similar to those seen in control WT cells. (Fig. 3a).

Regarding TJ proteins, claudin-5 and occludin immunostainings delineated endothelial cell borders as continuous lines in each experimental group (Fig. 3b, c). The intensity of claudin-5 immunofluorescence was similar in WT and APOB-100 transgenic endothelial cells under control conditions. A dramatic decrease in claudin-5 immunofluorescence intensity was observed following IL-6 in transgenic cells compared to control cells. This change was prevented by combining IL-6 with IL-10. Endothelial cells from WT mice showed no change in claudin-5 intensity after IL-6 exposure, but a significant increase in fluorescence intensity was detected following IL-10 treatment (Fig. 3b). The intensity of occludin immunofluorescence was similar in endothelial cells from both genotypes under control conditions. An increased fluorescence intensity was measured in WT cells following IL-6 and IL-10 treatments, but this effect was not detected after applying IL-6 and IL-10 in combination. APOB-100 endothelial cells showed an increase in occludin immunofluorescence after IL-10 exposure and no change in occludin fluorescence intensity was observed following other cytokine treatments (Fig. 3c). ZO-1 immunolabeling was present characteristically not only in cell borders but in the cytoplasm too, under control conditions especially in transgenic cells. Following each cytokine treatment, the cytoplasmic localization of ZO-1 was abundant in both genotypes. The intensity of ZO-1 immunofluorescence labeling was higher in transgenic cells under basal conditions. A significant increase in immunofluorescence intensity was seen in both genotypes after each cytokine application (Fig. 3d).

**Fig. 4:**
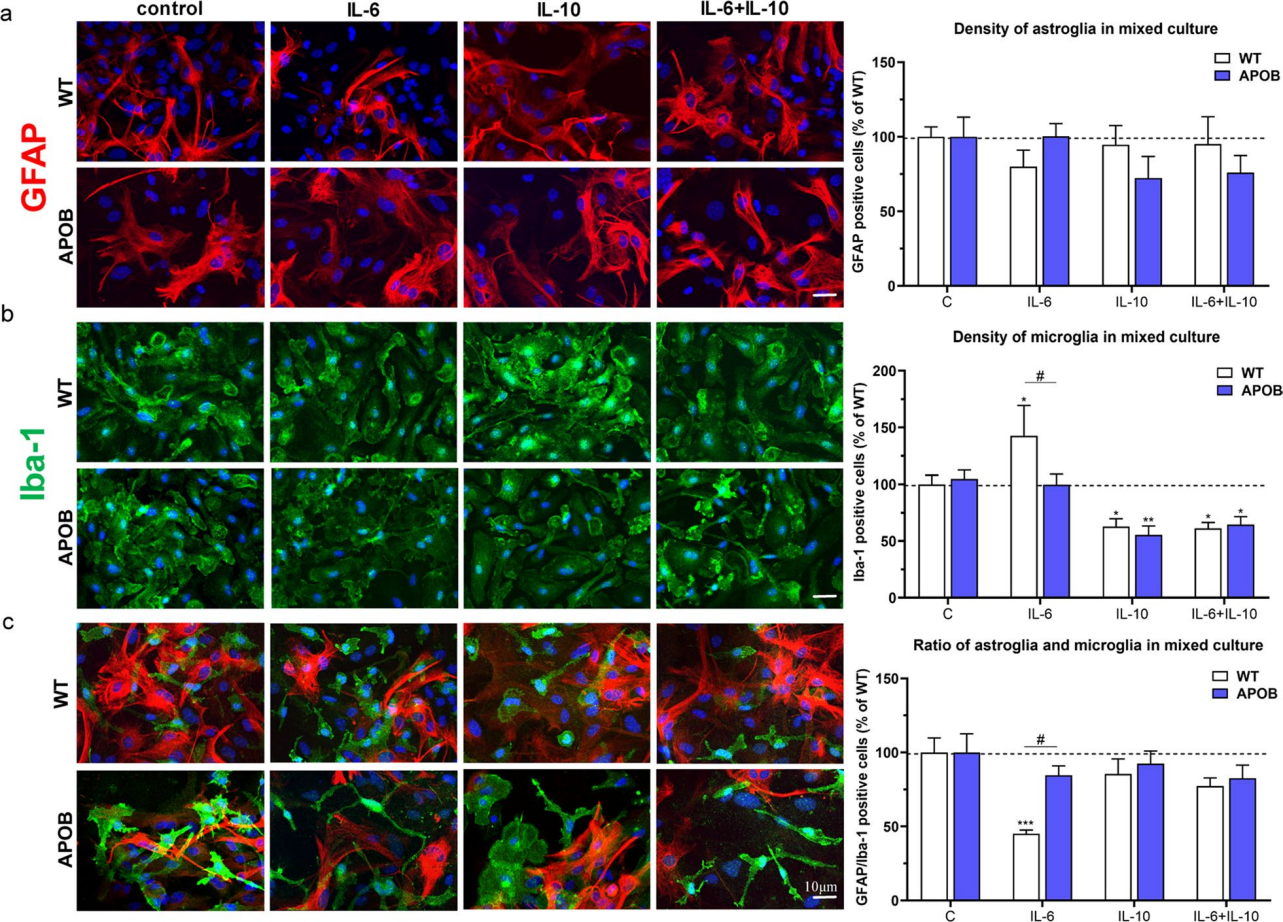
Changes in wild type (WT) and apolipoprotein B-100 (APOB-100) glial cell densities following cytokine treatments. Effects of IL-6, IL-10 and IL-6 + IL-10 cytokine treatments in primary glial cell cultures isolated from WT and APOB-100 transgenic mice on **a** astroglia density immunostained for GFAP, **b** microglia density immunostained for Iba-1, **c** astroglia/microglia ratio. #: significant change between WT and APOB-100 cells (p < 0.05); *: significant change due to cytokine treatment compared to the control group with the same genotype (p < 0.05)

In our next series of experiments other cellular components of the neurovascular unit, namely astro- and microglial cells were studied in vitro. Mixed astro/microglia cultures are suitable tools to study changes in astro- and microglia density and in the ratio of astro/microglial cells, which may reproduce glial reactions occurring in vivo. Astrocytes showed similar densities under control conditions and in all cytokine treated groups in both genotypes (Fig. 4a). The density of microglial cells without cytokine treatment was also similar comparing the two genotypes. Following IL-6 treatment WT microglia showed an increase in density. In contrast, after IL-10 exposure, applied either alone or in combination with IL-6, a decreased microglia density was observed in both genotypes, compared to their respective controls (Fig. 4b). The astro/microglia ratio under control conditions was also similar in APOB-100 transgenic and WT mixed glial cell cultures. In APOB-100 glial cell cultures no change in astro/microglia ratio was measured following either cytokine treatment. In contrast, in WT cultures a significant decrease in astro/microglia ratio was measured following IL-6 treatment. This effect was not seen when IL-6 and IL-10 were applied in combination. IL-10 alone did not have any effect on astro/microglia ratio in WT glial cells. (Fig. 4c).

**Fig. 5:**
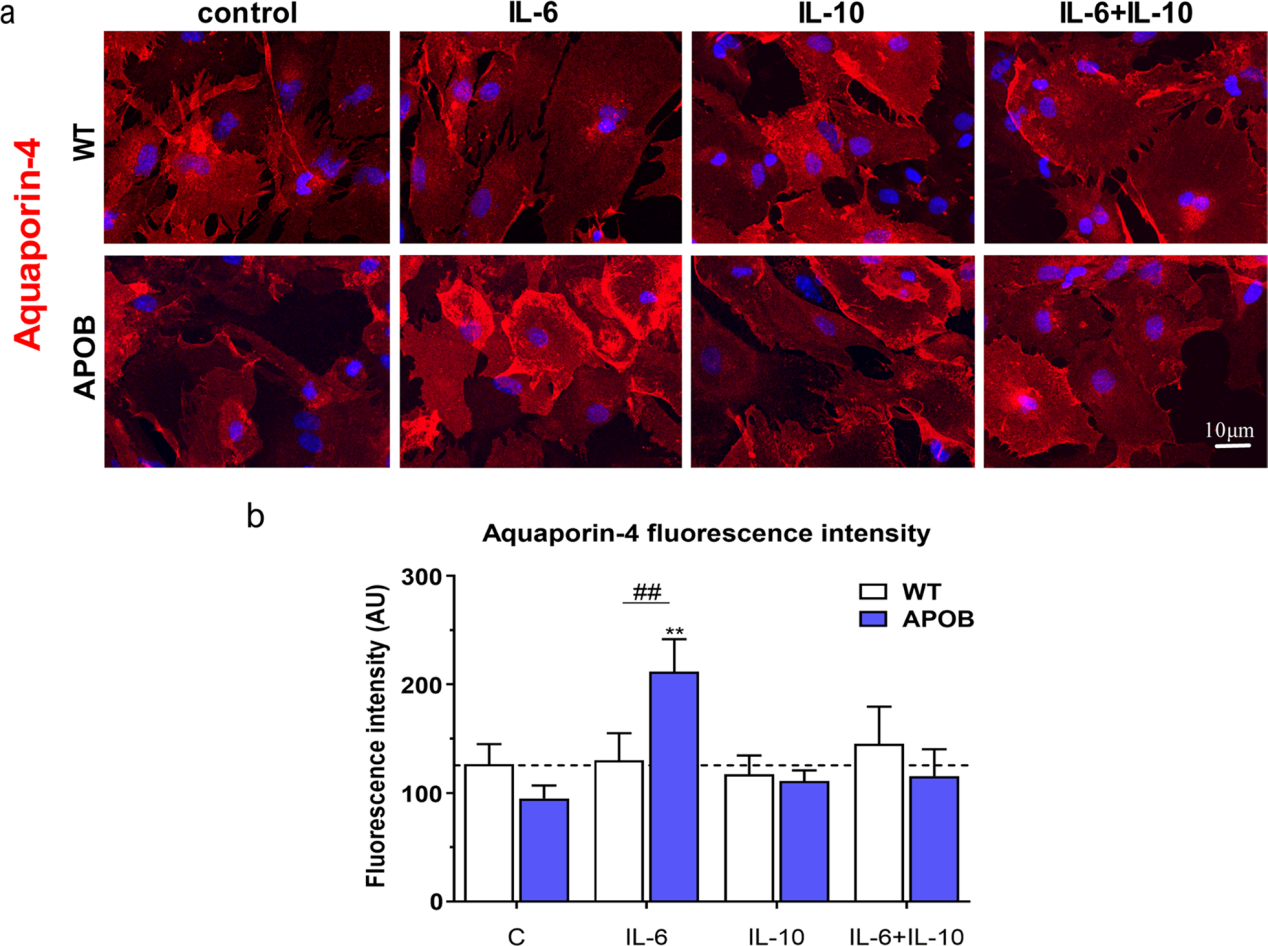
Changes in aquaporin-4 (a) immunostaining pattern and (b) fluorescence intensity in cultured astroglial cells. The cells were isolated from wild type (WT) and apolipoprotein B-100 (APOB-100) transgenic mice and treated with IL-6, IL-10 and IL-6 + IL-10 cytokines. AU: arbitrary unit. #: significant change between WT and APOB-100 cells (p < 0.05); *: significant change due to cytokine treatment compared to the control group with the same genotype (p < 0.05)

The characteristic protein of astroglial endfeet, the water channel AQP4, was examined in cultured astrocytes isolated from WT and APOB-100 mice (Fig. 5). The specificity of AQP4 immunolabeling was confirmed by its colocalization with the astroglia marker S100b (Additional file 1: Fig. S2). AQP4 immunofluorescence intensity was similar in primary astroglia cultures comparing the two genotypes under basal conditions. A significant increase in the intensity of AQP4 immunofluorescence was observed in APOB-100 transgenic astrocytes following IL-6 exposure. This effect was not seen when IL-6 was applied in combination with IL-10. No change in AQP4 fluorescence intensity was detected in transgenic astroglia when they were treated with IL-10 alone. AQP4 immunofluorescence intensity showed no change in primary astroglia cultures isolated from WT mice after either cytokine treatment (Fig. 5).

### Microvessels

**Fig. 6:**
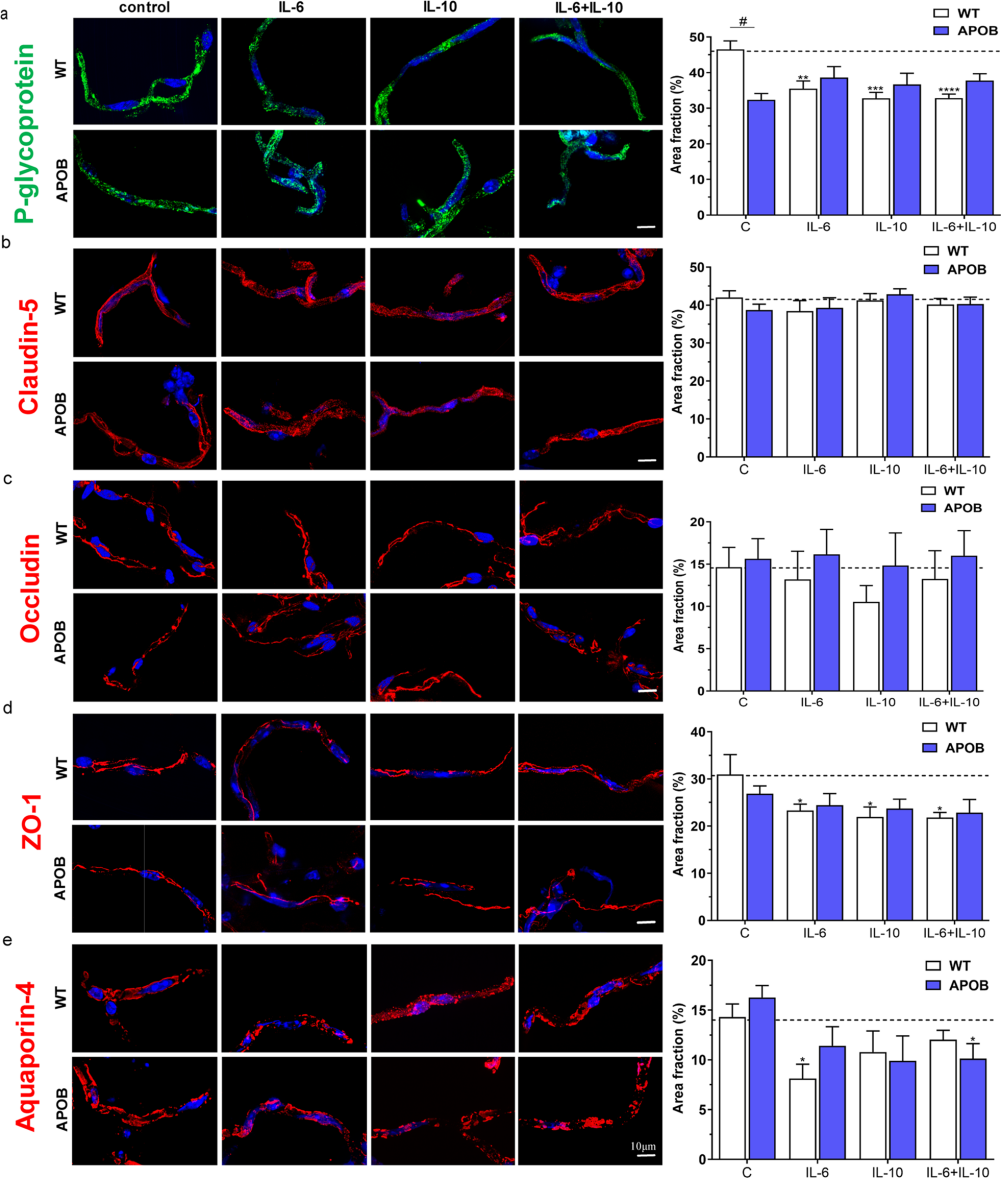
Changes of endothelial and astroglial cell markers in isolated brain microvessels. Microvessels were isolated from wild type (WT) and apolipoprotein B-100 (APOB-100) transgenic mice, treated with IL-6, IL-10 and IL-6 + IL-10 cytokines and immunostained for **a** P-glycoprotein, **b** claudin-5, **c** occludin, **d** zonula occludens protein-1 (ZO-1), **e** aquaporin-4. #: significant change between WT and APOB-100 (p < 0.05); *: significant change due to cytokine treatment compared to the control group with the same genotype (p < 0.05)

Morphological analysis of P-gp and TJ proteins expressed by endothelial cells and of AQP4 localized mainly in astroglial endfeet was carried out in isolated brain microvessels too (Fig. 6). The immunostaining of the efflux pump P-gp in isolated microvessels displayed an inhomogeneous pattern with dense patches in parts in the WT control group. In microvessels isolated from APOB-100 transgenic mice and in all cytokine treated WT microvessels the P-gp immunoreactivity pattern showed a more patchy appearance. The area fraction of P-gp labeling was significantly higher in WT microvessels compared to transgenics under basal conditions (Fig. 6a). Microvessels isolated from APOB-100 transgenic mice showed no change in the pattern of P-gp immunostaining following cytokine treatments. In contrast, a decrease in P-gp immunolabeled area fraction was observed in WT microvessels after each cytokine exposure (Fig. 6a).

Claudin-5 and occludin immunostained area fractions were similar in WT and APOB-100 transgenic microvessels without cytokine treatment. No change in these immunostained area fractions was detected after cytokine exposures (Fig. 6b, c). ZO-1 immunostaining, like other TJ proteins, showed no significant difference in immunolabeled area fraction in microvessels isolated from WT and APOB-100 transgenic animals. Transgenic microvessels were not responsive to cytokine treatments. In contrast, a decrease in immunolabeled area fraction was measured in WT microvessel after each cytokine application (Fig. 6d).

The immunolabeling of the astrocyte endfeet marker AQP4 was characterized by a similar area fraction in WT and APOB-100 microvessels without cytokine treatment. Application of the pro-inflammatory cytokine IL-6 resulted in a decrease in AQP4 immunostained area fraction compared to controls in WT microvessels. This effect was antagonized by IL-10 in WT microvessels, while IL-10 applied alone had no effect on AQP4 area fraction. In transgenic microvessels, in contrast, AQP4 area fraction did not change after IL-6 or IL-10 exposure, but showed a decrease when IL-6 was applied in combination with IL-10, and no antagonistic effect was observed (Fig. 6e).

## Discussion

### Cell cultures

The BBB dysfunction in the APOB-100 transgenic mouse model of human atherosclerosis was described in detail in our previous work [30]. In the present study we investigated the effects of IL-6 and its antagonist, IL-10, two cytokines related to atherosclerosis, to reveal their roles in BBB dysfunction and morphological changes using primary brain endothelial cell cultures and brain microvessels isolated from WT and APOB-100 transgenic mice.

**Fig. 7:**
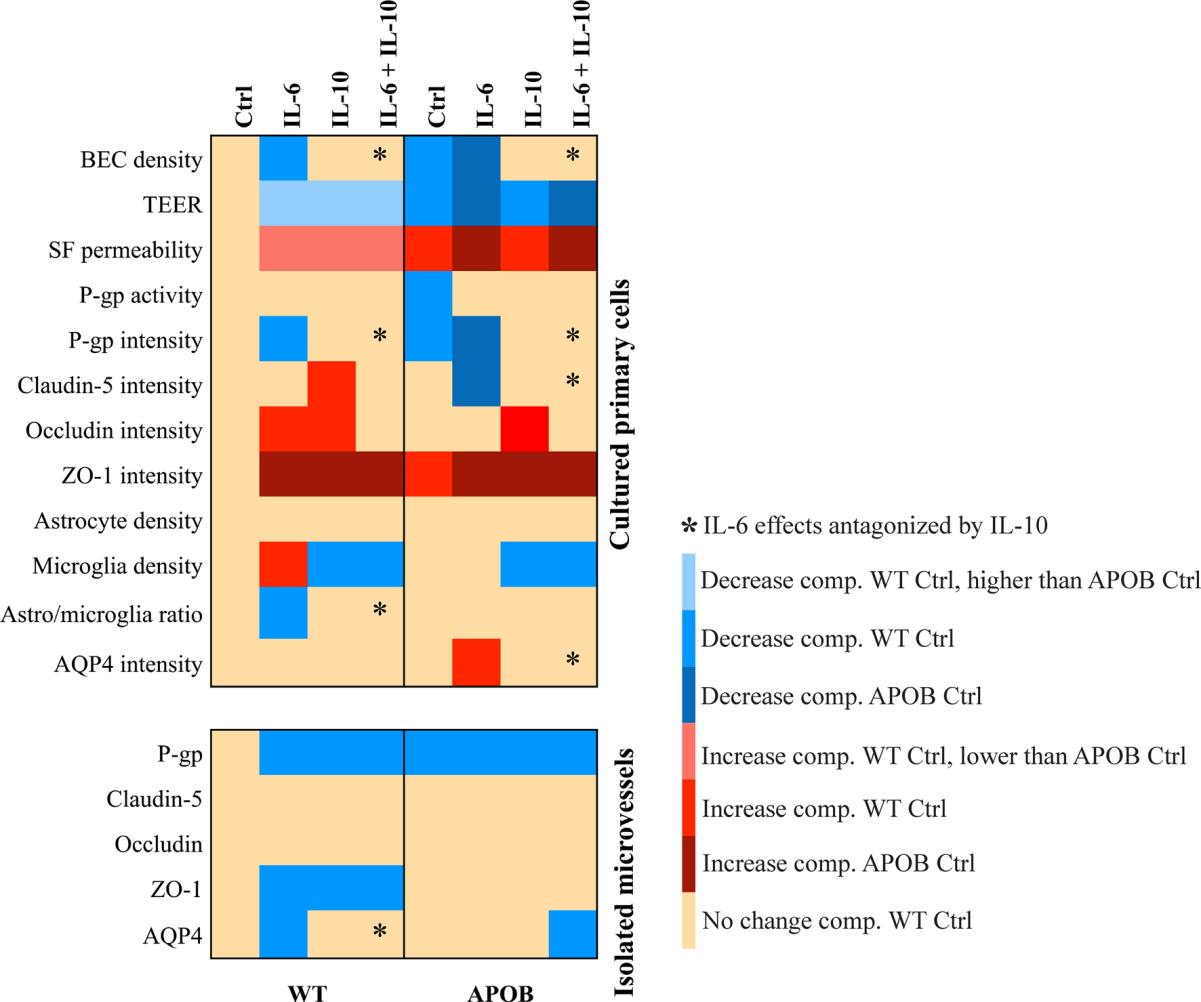
Summary of functional and morphological changes of BBB characteristics in wild type (WT) and apolipoprotein B-100 (APOB-100) models. Heat map representation of the effects of IL-6 and IL-10 cytokine treatments on BBB characteristics measured in WT and APOB-100 models

Under basal, unstimulated culture conditions APOB-100 brain endothelial cells grew slower than WT cells, which was also reflected in lower cell density. This difference in cell growth may, at least in part, be a consequence of the expression of the APOB-100 transgene in cultured brain endothelial cells, as we demonstrated previously [32], and the proinflammatory microenvironment of the cerebral microvessels from which endothelial cells were isolated [29, 30]. This is in line with the present finding that IL-6 gene expression was significantly higher in microvessels than in brain tissue in APOB-100 transgenic animals. Thus, cultured APOB-100 brain endothelial cells could be primed with IL-6 without further cytokine treatments. IL-10 applied alone did not influence either cell viability or cell density, but it antagonized IL-6 effects on both parameters when applied in combination with IL-6 (Fig. 7). This is in concordance with data showing that IL-10 downregulates inflammatory genes and antagonizes IL-6 actions [39, 40].

As compared to the WT, the APOB-100 BBB model showed a paracellular barrier dysfunction based on the decreased TEER values and enhanced permeability for SF under control conditions (Fig. 7). We measured increased BBB permeability in APOB-100 transgenic animals earlier [30], which supports the present observation. IL-6 is known to induce a dose- and time-dependent decrease in TEER in rat brain endothelial cell monocultures [41]. In accordance with this, IL-6 decreased TEER and increased paracellular permeability in both WT and APOB-100 BBB co-culture models in our present experiments (Fig. 7). Effects of IL-10 on in vitro BBB function were examined for the first time in our present experiments. We observed a decrease in TEER and an increase in SF permeability in the WT BBB co-culture model. APOB-100 transgenic brain endothelial cells, which are characterized by weaker paracellular barrier properties in control conditions, showed no change in these parameters following IL-10 treatment. The alterations in TEER and SF permeability seen after IL-6 application were not antagonized by IL-10 in either genotype (Fig. 7). Our data may result from a difference in cytokine expression in the WT compared to the APOB-100 brain. APOB-100 brain endothelial cells are isolated from an inflamed condition. Consequently, these cells are sensitive to treatment with the pro-inflammatory cytokine IL-6, but the anti-inflammatory IL-10 exposure is not harmful for them. In contrast, exposing WT brain endothelial cells either to IL-6 or IL-10 may result in an imbalance in their microenvironment, leading to decreased TEER and increased permeability values.

Our present results show that P-gp activity, another feature of BBB function, decreased in APOB-100 brain endothelial cells compared to WT cells without cytokine treatment (Fig. 7). Data on P-gp activity measured in vivo in neuroinflammatory conditions are reported in relation to neurodegenerative diseases. P-gp is overexpressed in epilepsy [42] and amyotrophic lateral sclerosis [43], and downregulated in Parkinson’s and Alzheimer’s diseases, where it leads to impaired β-amyloid clearance [16]. Under basal conditions P-gp activity in APOB-100 brain endothelial cells was reduced similarly to results observed in Parkinson’s or Alzheimer’s diseases. Both IL-6 and IL-10 treatments resulted in an improved P-gp activity compared to control conditions in the transgenic cells, while the efflux pump activity did not change in WT brain endothelial cells (Fig. 7). The published findings related to IL-6 effects on P-gp function are contradictory. IL-6 reduced P-gp function in cultured guinea pig brain endothelial cells [44], while it did not change P-gp activity in a human BBB model [45]. IL-6 is involved in the pathogenesis of amyotrophic lateral sclerosis [46, 47], a pathologic condition where P-gp activity is increased [43]. The elevated P-gp activity in APOB-100 brain endothelial cells treated with IL-6 may reflect an increased cellular vulnerability. Regarding IL-10 action, the observed improvement of P-gp function in transgenic brain endothelial cells may indicate a compensatory mechanism against some inflammatory processes [39] going on in hypertriglyceridemic mice from which endothelial cells were derived. WT endothelial cells, in contrast, showed a more stable P-gp activity, which was not altered by either cytokine treatment at least in the concentrations applied. The decreased P-gp activity detected in cultured APOB-100 brain endothelial cells compared to WT cells was paralleled by a decrease in P-gp immunofluorescence intensity under basal conditions. These changes are in line with our earlier results showing a decrease in P-gp immunofluorescence intensity of microvessels in brain sections of APOB-100 transgenic mice [30]. The P-gp immunostaining pattern in our primary mouse brain microvessel endothelial cell cultures was similar to that seen in our previous works on rat [48, 49] and earlier publications on rat and bovine BBB models [50, 51]. Regarding IL-6 effects on P-gp protein expression, the published results tend to show a reduction in P-gp content after pro-inflammatory cytokine treatments [52, 53], which supports our present data (Fig. 7). IL-10, in contrast, resulted in an increase in P-gp immunofluorescence intensity in APOB-100 endothelial cells and antagonized IL-6 effects both in WT and APOB-100 brain endothelial cells. These data provide further support for the antagonistic actions of IL-6 and IL-10, and demonstrate for the first time that P-gp expression in brain microvascular endothelial cells may be enhanced by IL-10.

The major proteins determining TEER and paracellular permeability at the BBB are TJ proteins. An immunocytochemical analysis of TJ proteins in microvessel endothelial cells isolated from WT and APOB-100 transgenic mouse brains under basal conditions was described in our earlier publication [32]. In that study cytoplasmic linker ZO-1 showed a significant increase in fluorescence intensity in APOB-100 endothelial cells compared to WT cells, but no difference was found in integral membrane TJ proteins claudin-5 and occludin fluorescence intensity. Our present results partly confirm these earlier findings and provide new data on TJ protein expressional changes following IL-6 and IL-10 treatments. Among TJ proteins claudins are fundamental elements in the regulation of paracellular permeability and their expression is reduced due to inflammation [54]. The pro-inflammatory cytokine IL-6 reduced claudin-5 expression in human and in rat brain microvascular endothelial cell cultures [55, 56]. In our murine brain microvascular endothelial cell cultures a decrease in claudin-5 fluorescence intensity was detected in APOB-100 transgenic cells after IL-6 treatment, which was prevented by a simultaneous IL-10 application (Fig. 7). This observation is supported by data indicating that IL-10 attenuated the decrease in claudin-5 expression induced by the pro-inflammatory cytokine TNFα in rat brain endothelial cell cultures [56]. Furthermore, IL-10 attenuated the increase in BBB permeability and the downregulation of claudin-5 in a rat model of severe acute pancreatitis [57]. WT endothelial cells, in contrast, showed no change in claudin-5 fluorescence intensity after IL-6 treatment. This suggests a possible difference in sensitivity to pro-inflammatory cytokines of brain endothelial cells in different species and strains within species. An increased sensitivity to IL-6 in APOB-100 transgenic compared to WT cells can also be concluded (Fig. 7). Following IL-10 exposure an increase in claudin-5 immunostaining intensity measured in all cell areas including the cytoplasm was observed in WT brain endothelial cell cultures. This is in line with our results showing a decrease in TEER and an increase in paracellular permeability in WT endothelial cells treated with IL-10, indicating that a balanced cytokine microenvironment is needed for optimal claudin-5 expression, localization and BBB function.

Occludin fluorescence intensity showed similar values in WT and APOB-100 endothelial cells without cytokine treatment (Fig. 7), which confirmed our earlier results [32]. An increase in the intensity of occludin immunofluorescence was detected in WT brain endothelial cells after IL-6 and IL-10 treatments and in APOB-100 brain endothelial cells following IL-10 application. Occludin is reported to undergo a continuous endocytosis from the plasma membrane into the cytoplasm, followed by a recycling back to the plasma membrane [58]. A possible regulatory mechanism of occludin turnover involves glycogen synthase kinase 3β (GSK3β), a ubiquitously expressed serine/threonine kinase having multiple functions. GSK3β was downregulated in IL-6 treated hepatocytes [59]. Inhibition of GSK3β resulted in increased occludin levels in brain endothelial cells based on western blot studies [60]. This is a possible mechanism that may explain the increase in occludin fluorescence measured in WT endothelial cells after IL-6 exposure, while the GSK3β in APOB-100 endothelial cells may be less sensitive to IL-6. On the other hand, occludin endocytosis may be enhanced by IL-10, since IL-10 is capable of inducing actin filament rearrangements leading to endocytosis [61]. IL-10 may promote endocytosis in both WT and APOB-100 endothelial cells, contributing to occludin internalization, which may result in an increase in occludin immunofluorescence intensity. Furthermore, a recent study found that occludin increase may compensate for a loss in claudin-5 at the BBB in claudin-5 null mouse embryo [62].

Another TJ protein examined in the present study is ZO-1, which participates in actomyosin organization, cell–cell tension, cell migration, angiogenesis and barrier formation in endothelial cells [63]. In our models an increase in ZO-1 immunofluorescence intensity was observed in APOB-100 transgenic endothelial cells compared to WT cells under control conditions and in both genotypes following all cytokine treatments (Fig. 7) most possibly due to changes in ZO-1 intracellular distribution. Our observations are supported by findings that report a decrease in ZO-1 mRNA levels and a redistribution of ZO-1 protein from the cell borders toward cellular nuclei after IL-6 application in human brain microvascular endothelial cell cultures [64]. ZO-1 relocalization was also seen in brain endothelial cells in a mouse model of multiple sclerosis [65]. IL-10 exerted similar effects as IL-6 on changes in ZO-1 immunofluorescence intensity.

Our in vitro experiments focused not only on endothelial cells, but on glial components of the neurovascular unit, too. First, changes in cell density of cultured astro- and microglia were analyzed to examine whether IL-6 applied at the concentration of 50 ng/ml in endothelial cell cultures was also effective in our glial cell cultures. The observed increase in WT microglia density served as a proof of effectiveness of the IL-6 concentration in cultured glial cells (Fig. 7). It suggests an increase in microglia proliferation after IL-6 application, which is in line with data describing similar IL-6 effects [66]. The lack of reactivity to IL-6 treatment in APOB-100 transgenic microglia may result from a decreased sensitivity to IL-6, since these cells are derived from chronic neuroinflammatory conditions. Chronic exposure to IL-6 is reported to induce a desensitized microglia phenotype [67]. IL-10, in contrast, is known to reduce microglia proliferation [68, 69], which confirms our present results seen both in WT and APOB-100 glial cell cultures. Our data suggest that IL-10 antagonizes IL-6 effects on density in both WT and APOB-100 microglia cells. Regarding astrocytes, in our present experiments no significant changes were detected in astroglia density at 24 h following cytokine treatments. There are conflicting data on IL-6 effects on astroglia proliferation rate [70, 71], while IL-10 is unanimously reported to promote proliferation of astroglia [72]. The lack of reactivity to cytokine treatments in our cultured astrocytes at 24 h indicates that astroglia proliferate in response to stimuli later then microglia as in vivo. The changes occurring in parallel in micro- and astroglial cells in our mixed cultures resulted in a significant decrease in the astro-/microglia ratio in WT glial cultures following IL-6 treatment which was antagonized by IL-10 (Fig. 7).

The water channel AQP4, a marker protein of astroglial endfeet, was analyzed in WT and APOB-100 astroglia cultures. AQP4 upregulation is reported during neuroinflammation [73] and IL-6 is a known driving force in this process [74]. Our present results showing an increase in AQP4 fluorescence intensity following IL-6 treatment in cultured APOB-100 astrocytes (Fig. 7) are in accordance with these data, and demonstrate that IL-6 effects on AQP4 expression in APOB-100 astrocytes may be dependent on the inflammatory microenvironment. No change in AQP4 fluorescence intensity was seen in the WT group following IL-6. The anti-inflammatory cytokine IL-10 antagonized IL-6 actions on AQP4 fluorescence intensity changes in APOB-100 astroglial cells.

### Microvessels

We examined cytokine effects on immunostaining patterns of key proteins playing a role in BBB function not only in cell cultures, but also in isolated brain microvessels. This ex vivo system has more similarity to in vivo conditions, since in these isolated microvessels endothelial cells are surrounded by pericytes [35] and astrocytic endfeet. Consequently, cellular interactions may modulate the effects of cytokine treatments. To preserve the ex vivo function of isolated brain microvessels, cytokine treatments lasted for one hour, in contrast to the one-day treatment length of cell cultures.

P-gp immunolabeling in isolated brain microvessels showed a reduction in the transgenic group compared to WT brain microvessels under control conditions as described in our earlier publication using brain sections [30]. This decreased P-gp staining was also observed in primary brain endothelial cell cultures, as shown in Fig. 7. The reactivity to cytokines, however, was different in microvessels compared to endothelial cell monocultures. In APOB-100 microvessels, characterized by a reduced P-gp immunolabeling, no further decrease to cytokine treatments was seen. This lack of reactivity to IL-6 and IL-10 application may result from the increased IL-6 production in APOB-100 microvessels under basal conditions, as it was observed in our qPCR experiments (Fig. 1). WT microvessels, in contrast, showed a decrease in area fraction of P-gp immunolabeled structures in each cytokine treated group. It resulted in a P-gp immunolabeling pattern similar to that seen in transgenic microvessels (Fig. 6). Astrocytic endfeet and pericytes, which are present in isolated microvessels around endothelial cells, are known to regulate endothelial cell polarity, function and P-gp localization [75, 76]. Moreover, both astrocytes and pericytes are capable to secrete IL-6 and IL-10 [77], and astrocytes themselves are responsive to both IL-6 and IL-10 [78, 79]. The possibility of a complex cellular interaction and reactivity to cytokines in isolated microvessels may explain the differences detected in P-gp immunolabeling in isolated microvessels compared to brain endothelial cell monocultures.

In contrast to brain endothelial cell monocultures, isolated brain microvessels showed no change in claudin-5 and occludin immunostaining after 1-h cytokine exposures (Fig. 7). IL-6 is reported to induce a decrease in claudin-5 and occludin protein expression in microvessels from adult sheep at a concentration of 100 ng/ml, but no change was detected in these protein levels when IL-6 was applied at 10 ng/ml [80]. This indicates that IL-6 effects on claudin-5 and occludin expression are dose dependent in microvessels. In the present paper we used IL-6 at a concentration of 50 ng/ml, which might not be a concentration high enough to affect claudin-5 and occludin expression.

ZO-1, another TJ protein analyzed in our experiments, showed a decrease in ZO-1 immunolabeled area fraction in WT microvessels following each cytokine treatment (Fig. 7). In a previous study we also demonstrated a reduced ZO-1 immunolabeling in isolated rat brain microvessels in inflammatory condition [35]. ZO-1 immunoreactive area fraction in APOB-100 microvessels, in contrast to the WT, remained the same after each cytokine treatment as in control conditions. A similar decrease in sensitivity to IL-6 and IL-10 application was detected in P-gp immunoreactive area fraction in the transgenic microvessels, suggesting that P-gp and ZO-1 immunoreactivity may share some regulatory features. The differences seen in ZO-1 changes in brain endothelial cells in culture and in isolated microvessels may partly be due to differences in cytokine treatment times in the two systems.

In addition to TJ proteins characteristic to cerebral endothelial cells, the immunostaining pattern of AQP4, a specific component of astrocytic endfeet, was also analyzed in isolated brain microvessels. Under basal conditions no difference was seen between AQP4 area fractions measured in WT and APOB-100 microvessels. However, the sensitivity to cytokine treatments was different comparing the two genotypes. WT microvessels reacted to IL-6 treatment only, while microvessels isolated from transgenic mice showed a decrease in AQP4 immunolabeled area fraction following a combined IL-6 + IL-10 exposure. Decreases in the AQP4 immunolabeled area fractions in isolated brain microvessels may suggest either reductions in AQP4 protein expression or relocalization of the AQP4 protein in astrocytic endfeet. Our previous study analyzing brain capillary ultrastructure in APOB-100 transgenic mice indicated edema of astroglia endfeet, which was in line with an increased expression of the AQP4 gene in isolated microvessels [30]. Therefore, we hypothesize that the observed decreases in AQP4 area fractions are the consequences of a disturbance in the normal localization of the water channel AQP4. Mislocalization of AQP4 was reported in epilepsy and a loss of AQP4 in astroglial endfeet was found in mouse models of Alzheimer’s disease and multiple sclerosis indicating BBB impairment [75, 81, 82]. In isolated brain microvessels the principal cellular targets of IL-6 and IL-10 are endothelial cells and/or pericytes, which can, in turn, affect AQP4 localization in astroglial endfeet via intercellular signaling. Treating isolated microvessels with IL-6 may mimic neuroinflammatory conditions during which AQP4 is decreased in astrocytic endfeet. A possible route of IL-6 action is the activation of the NF-κB pathway in the wall of microvessels [74, 83] which is inhibited by the anti-inflammatory cytokine IL-10 [84]. Consequently, IL-10 may antagonize IL-6 effects on AQP4 immunoreactivity pattern, as it was detected in WT microvessels (Fig. 7). APOB-100 microvessels, in contrast, did not show a reduction in AQP4 immunoreactive area fraction following IL-6. However, a decrease in AQP4 area fraction was seen after a combined IL-6 + IL-10 action. It may indicate that transgenic microvessels are less sensitive to IL-6 than WT microvessels when IL-6 is applied alone. It also suggests that in transgenic microvessels instead of the NF-κB pathway IL-6 and IL-10 may use other signaling pathways, such as the transcription factor STAT3. Both IL-6 and IL-10 can activate STAT3 in brain endothelial cells [57, 85], which may explain the lack of antagonism between IL-6 and IL-10 in APOB-100 microvessels.

Our study has certain limitations. One of them is that a large part of the study is morphological which can by analyzed semi-quantitatively. Analysis of the possible molecular pathways leading to the observed cytokine actions was beyond the scope of our study. Investigating the involvement of IL-6 and IL-10 in the activation of STAT3 and NF-κB pathways and in the regulation of endocytosis via GSK3β inhibition or actin filament rearrangements could provide a deeper understanding of the increased BBB permeability seen in hypertriglyceridemia. The cell specificity of the underlying molecular mechanisms should also be taken into account and the possible modulatory effects of cellular interactions should be explored in order to define the key elements of IL-6 and IL-10 actions at the BBB.

Taken together, we demonstrated functional and morphological differences between WT and APOB-100 brain endothelial cells under control conditions (Fig. 7). Functional characteristics, such as TEER, SF permeability and P-gp activity were sensitive to both IL-6 and IL-10 cytokine treatments, but no antagonistic effect was observed. In contrast, a decrease in brain endothelial cell density and P-gp immunofluorescence intensity, which were detected in both genotypes following IL-6 treatment, were antagonized by IL-10. Other BBB features sensitive to IL-6 in APOB-100 brain endothelial cells included a decrease in claudin-5 and an increase in AQP4 fluorescence intensity, and a decrease in astro-/microglia ratio in WT glia cultures. These IL-6 induced changes were also antagonized by IL-10. Isolated brain microvessels in general, and APOB-100 microvessels in particular, were less reactive to cytokine treatments than cell cultures. In this ex vivo system IL-6 resulted in a decrease in P-gp, ZO-1 and AQP4 immunostained area fractions in WT microvessels. The decrease in AQP4 immunolabeled area fraction was antagonized by IL-10. Following treatment with the anti-inflammatory cytokine IL-10 a decrease in P-gp and ZO-1 immunolabeled area fraction was seen, and no antagonistic effect was observed between IL-6 and IL-10 action regarding P-gp and ZO-1 changes in WT microvessels. Our present results identify BBB characteristics sensitive to either IL-6 or IL-10 actions, and demonstrate for the first time that IL-10 can prevent, at least in part, IL-6 induced BBB impairment.

## Supplementary Information

**Additional file 1.** Additional information regarding impedance kinetics, specificity of AQP4 immunocytochemistry and list of antibodies and gene specific primers used in this study.

## Data Availability

The dataset used and/or analysed during the current study are available from the corresponding author on reasonable request.

## Abbreviations

APOB: Apolipoprotein B
AQP4: Aquaporin 4
BBB: Blood–brain barrier
BEC: Brain endothelial cell
BSA: Bovine serum albumin
DMEM: Dulbecco’s modified Eagle’s medium
IL: Interleukin
LDL: Low-density lipoprotein
PBS: Phosphate buffered saline
P-gp: P-glycoprotein
SF: Sodium fluorescein
TEER: Transendothelial electrical resistance
TJ: Tight junction
TNF: Tumor necrosis factor
WT: Wild type
ZO-1: Zonula occludens-1
