# Supplementary material for: Role of interleukin-6 and interleukin-10 in morphological and functional changes of the blood–brain barrier in hypertriglyceridemia
Source: Fluids Barriers CNS. 2023 Mar 7;20:15. doi: 10.1186/s12987-023-00418-3 (PMC9990353; doi:10.1186/s12987-023-00418-3)
Supplement: Supplementary file 1 — Additional file 1. Additional information regarding impedance kinetics, specificity of AQP4 immunocytochemistry and list of antibodies and gene specific primers used in this study. [file 12987_2023_418_MOESM1_ESM.pdf]

Additional file 1

Role of interleukin-6 and interleukin-10 in morphological and functional changes of the blood-brain barrier in hypertriglyceridemia

Beáta Barabási, Lilla Barna, Ana Raquel Santa-Maria, András Harazin, Réka Molnár, András Kincses, Judit P. Vigh, Brigitta Dukay, Miklós Sántha, Melinda E. Tóth, Fruzsina R. Walter, Mária A. Deli, Zsófia Hoyk

Figure S1

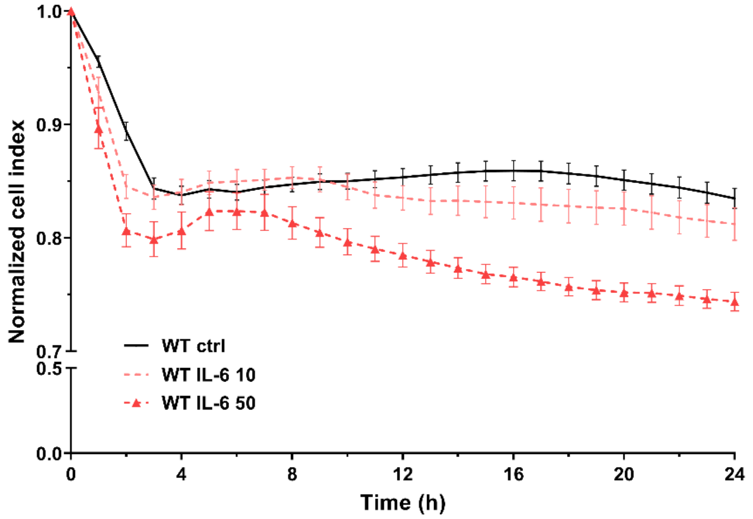

Concentration and time dependent effects of IL-6 on wild type primary mouse brain endothelial cell viability measured by impedance kinetics.

Figure S2

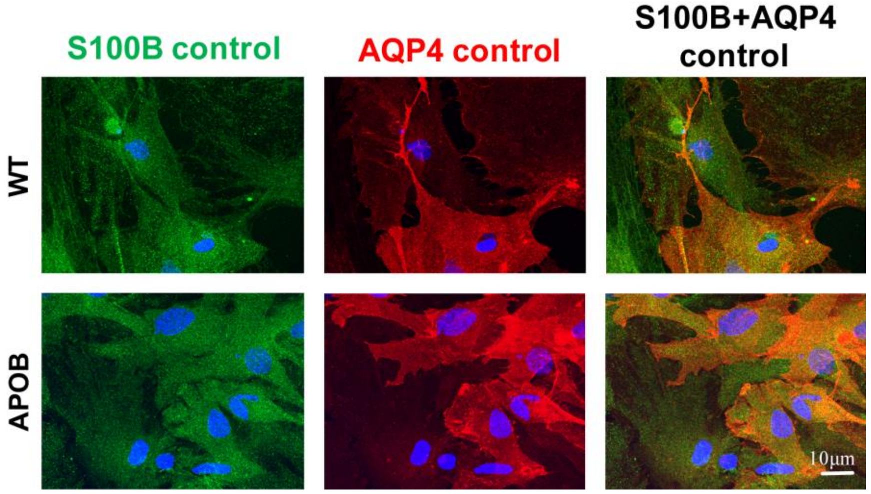

Immunostaining for astroglial cell markers S100B and aquaporin-4 (AQP4) in cultured glial cells. The cells were isolated from wild type (WT) and apolipoprotein B-100 (APOB-100) transgenic mice. AQP4 staining labels astroglial cells positive for S100B. Scale bar: 10 µm.

Table S1. Gene-specific primers for qPCR analysis

	gene	forward primer	reverse primer
1	*Gapdh*	GGGTTCCTATAAATACGGACTGC	CCATTTTGTCTACGGGACGA
2	*Actb*	CTAAGGCCAACCGTGAAAAG	ACCAGAGGCATACAGGGACA
5	*Il6*	GCTACCAAACTGGATATAATCAGGA	CCAGGTAGCTATGGTACTCCAGAA
6	*Il10*	CAGAGCCACATGCTCCTAGA	TGTCCAGCTGGTCCTTTGTT

Table S2. List of antibodies used in this study
A: Alexa Fluor

Antibody name	Vendor	Catalogue number	Host organism	Concentrations	Antibody registry ID
**Fluorescent immunostaining - Glia cultures (culture purity assessment)**					
anti-GFAP	Sigma	G3893	mouse	12.17 µg/ml	AB_477010
anti-Iba1	Abcam	ab5076	goat	0.5 µg/ml	AB_2224402
A488 anti-mouse	Invitrogen	A21202	donkey	1 µg/ml	AB_141607
A488 anti-goat	Invitrogen	A11055	donkey	1 µg/ml	AB_2534102
Dylight 549 anti-mouse	Jackson ImmunoResearch	115-505-003	goat	0.94 µg/ml	-
**Fluorescent immunostaining - Cell cultures**					
anti-claudin	Sigma	SAB4502981	rabbit	0.625 µg/ml	AB_10753223
anti-occludin	Invitrogen	71-1500	rabbit	0.2 µg/ml	AB_2533977
anti-ZO1	Invitrogen	61-7300	rabbit	0.25 µg/ml	AB_2533938
anti-Pgp	Calbiochem	517310	mouse	0.4 µg/ml	AB_564389
anti-Aqp4	Merck	AB3594	rabbit	0.4 µg/ml	AB_91530
anti-Iba1	Abcam	ab5076	goat	0.5 µg/ml	AB_2224402
anti-GFAP	Sigma	G3893	mouse	3.65 µg/ml	AB_477010
anti-S100B	Synaptic System	287011	mouse	0.6 µg/ml	AB_2814881
A594 anti-rabbit	Invitrogen	A21207	donkey	1 µg/ml	AB_141637
A488 anti-mouse	Invitrogen	A11029	goat	1 µg/ml	AB_2534088
Cy3-anti-rabbit	Sigma	C2306	sheep	2.5 µg/ml	AB_258792
A488 anti-goat	Invitrogen	A11055	donkey	1 µg/ml	AB_2534102
DyLight 549 anti-mouse	Jackson ImmunoResearch	115-505-003	goat	0.94 µg/ml	-
